# Rehabilitation of Upper Limb in Children with Acquired Brain Injury: A Preliminary Comparative Study

**DOI:** 10.1155/2018/4208492

**Published:** 2018-03-14

**Authors:** Elena Beretta, Ambra Cesareo, Emilia Biffi, Carolyn Schafer, Sara Galbiati, Sandra Strazzer

**Affiliations:** ^1^Scientific Institute IRCCS Eugenio Medea, Bosisio Parini, Lecco, Italy; ^2^Dipartimento di Elettronica, Informazione e Bioingegneria, Politecnico di Milano, Milan, Italy; ^3^School of Biomedical Engineering, Science and Health Systems, Drexel University, Philadelphia, PA, USA

## Abstract

Acquired brain injuries (ABIs) can lead to a wide range of impairments, including weakness or paralysis on one side of the body known as hemiplegia. In hemiplegic patients, the rehabilitation of the upper limb skills is crucial, because the recovery has an immediate impact on patient quality of life. For this reason, several treatments were developed to flank physical therapy (PT) and improve functional recovery of the upper limbs. Among them, Constraint-Induced Movement Therapy (CIMT) and robot-aided therapy have shown interesting potentialities in the rehabilitation of the hemiplegic upper limb. Nevertheless, there is a lack of quantitative evaluations of effectiveness in a standard clinical setting, especially in children, as well as a lack of direct comparative studies between these therapeutic techniques. In this study, a group of 18 children and adolescents with hemiplegia was enrolled and underwent intensive rehabilitation treatment including PT and CIMT or Armeo®Spring therapy. The effects of the treatments were assessed using clinical functional scales and upper limb kinematic analysis during horizontal and vertical motor tasks. Results showed CIMT to be the most effective in terms of improved functional scales, while PT seemed to be the most significant in terms of kinematic variations. Specifically, PT resulted to have positive influence on distal movements while CIMT conveyed more changes in the proximal kinematics. Armeo treatment delivered improvements mainly in the vertical motor task, showing trends of progresses of the movement efficiency and reduction of compensatory movements of the shoulder with respect to other treatments. Therefore, every treatment gave advantages in a specific and different upper limb district. Therefore, results of this preliminary study may be of help to define the best rehabilitation treatment for each patient, depending on the goal, and may thus support clinical decision.

## 1. Introduction

Acquired brain injuries are nonprogressive, nonhereditary brain injuries acquired sometime after birth and are the leading cause of long-term disability and death in children and young adults. Resulting from trauma, hypoxia, stroke, infection, or a variety of other sources, ABIs can lead to a wide variety of impairments, including deficiencies in cognitive, behavioral, metabolic, motor, perceptual motor, and/or sensory brain functions. In particular, a number of ABIs lead to significant hemiplegia [1], a weakness or paralysis on one side of the body, with relevant effects on the upper limb functionality [2].

In cases of hemiplegia, the aim of rehabilitation of the upper limbs is to prevent the disuse of the impaired side of the body. Many studies in literature have shown that therapy involving sensorimotor exercises to simulate meaningful tasks used in daily life increases the functional recovery of the affected upper limb [3, 4]. Realistic contexts of functional activities, such as reaching or pointing towards an everyday object, help patients acquire control strategies to compensate for muscle weakness and inaccuracies [5].

In order to rehabilitate the upper limbs, there are a variety of treatments available. Physical therapy (hereafter PT) is standard practice worldwide, but there are other types. Therapy aided by a robotic exoskeleton is noted for its capability of supporting repetitive and high-intensity training tasks as well as its ability to reliably track patients' motor progress by quantitatively measuring patients' movement kinematics and forces, rather than relying on subjective impressions [6]. When combined with interactive programs such as virtual reality, robot-aided therapy can assign functional meaning to the therapy, creating a motivating environment [6–9]. Studies involving robotic therapy have shown improvements in upper limb coordination and fluency of movement in the hands and fingers of children with cerebral palsy [10, 11].

Constraint-Induced Movement Therapy (CIMT) involves constraining the nonimpaired upper extremity in order to encourage use of the impaired side. CIMT therapy often involves intensive repetitive tasks directed by a therapist in order to practice motor movements [12]. CIMT is a rehabilitative methodology widely used nowadays, even on infants below one year of age (named baby-CIMT); it is considered feasible and without adverse effects [13]. Studies involving CIMT had shown improvements in the reaction time and movement-path length and improved smoothness in actions of the impaired limb in adults, with the overall goal to increase functional use and support cortical reorganization [12, 14–19].

There are numerous standardized clinical functional scales used for the assessment and evaluation of upper limb impairment and activity limitation. The majority of these utilize an ordinal-level scoring system, with scores assigned to the patient by the observing physician or therapist. Another way to assess upper limb activity is through kinematic data from 3D motion capture. Kinematic data is more objective and quantifiable, and there are a myriad of metrics available for calculation, both temporal, such as time and velocity, and spatial, such as joint angles and trunk displacement. However, the protocol for the measurement of kinematics is less standardized and can be difficult to compare, especially when dealing with child subjects [14].

Although previous studies have shown promising results of CIMT and robot-aided therapy in children [9, 12, 20], there is a lack of quantitative evaluations of effectiveness in a standard clinical setting as well as a lack of direct comparative studies between these therapeutic techniques.

The purpose of the present study is to quantify and compare the effects of constraint, robot-aided, and physical therapies in the rehabilitation of upper limbs of children and adolescents after ABI measured by both functional scales and kinematic data. Preliminary results have been presented in [21].

## 2. Materials and Methods

A group of children/adolescents with hemiplegia was enrolled in the study.

### 2.1. Subjects

In order to be included in this study, participants had to be 4–18 years old, had to have a clinical form of hemiplegia and a severe acquired brain injury, and had to have the ability to understand and follow test instructions. Patients were excluded from the study if they had severe muscle spasticity and/or contracture, a diagnosis of severe learning disabilities or behavioral problems, visual or hearing difficulties that would impact on function and participation, previously undergone restraint therapy, or injections of antispasticity drugs (e.g., Botox or Dysport) into the upper limb musculature in the 6 months leading up to the beginning of the trial.

Taking these criteria into consideration, eighteen children/adolescents (hereafter, participants) (9 M and 9 F, mean age: 12.28 ± 5.13 years) were recruited: ten had right-sided and eight had left-sided impairments. The mean age at injury was 10.95 (±4.88) years. Eight participants had a traumatic brain injury, seven participants had a stroke, two participants had a brain injury due to encephalitis, and one participant had a brain injury due to other causes. They took part in the clinical protocol 1.25 (±1.02) years after the injury.

All parents were informed about the study and signed a consent statement. The study was approved by the Ethics Committee of the Scientific Institute IRCCS Eugenio Medea, located in Bosisio Parini, Italy, in accordance with the declaration of Helsinki.

### 2.2. Rehabilitative Protocol

The rehabilitation program included two consecutive four-week periods of treatment, attended in a random order: one period of physical therapy (PT) and one period of a rehabilitative treatment, randomly chosen between Constraint-Induced Movement Therapy (CIMT) and training with Armeo®Spring.

PT treatment was administered in five 45-minute sessions per week for each of the four weeks. PT emphasized fine and gross motor skills and multimodal exploration, with the overall goal of successfully performing independent daily living skills such as self-care and eating. It was based on motor control and motor-learning theories, task-oriented and customized on the single patient's functional status. Fine motor skills included monomanual and bimanual grasping and use of individual fingers, while gross motor skills focused on reactive balance responses and postural support. There were four types of task goals: perceptual motor activities; activities of reaching, grasping, holding, and manipulating; activities for posture and balance; and self-care and daily living activities.

During Constraint-Induced Movement Therapy (CIMT), a restraining thermoplastic splint was worn on the unaffected hand, preventing subjects from flexing their fingers or grasping objects. Even though the thumb was locked in a fixed position against the index finger, children could use the hand for support or to break a fall. The splint was worn for at least 3 consecutive hours a day, every day of the week for 4 weeks. While the splint was worn, children/adolescents underwent an intensive rehabilitation program to simulate play sessions and a daily living activity. Unimanual activities performed included memory cards, puzzles, playing bowls and cards, using a spoon or fork, and/or dusting a surface.

**Figure 1:**
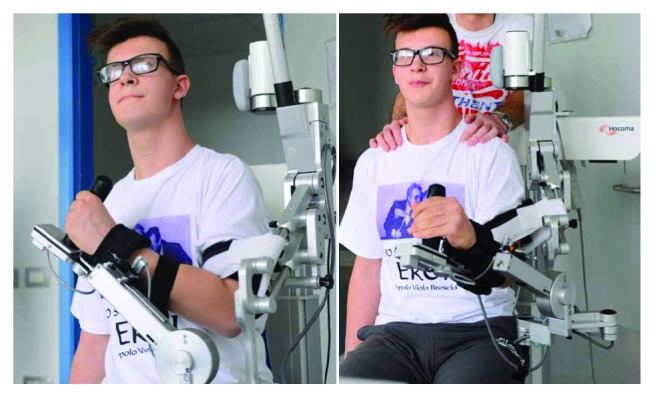
The exoskeleton Armeo®Spring.

Armeo®Spring is an exoskeleton with five degrees of freedom that uses springs (rather than robotic actuators) to guarantee passive arm weight support and guidance (Figure 1). By adapting to each patient's individual morphology and residual ability, the Armeo exoskeleton enables users to achieve a large range of motion in a 3D workspace. Subjects using Armeo were given 45-minute treatment sessions 5 times a week for 4 weeks. In each session, subjects used dedicated system software to simulate intense and meaningful tasks targeting different upper arm joints and regions. Physical therapists oversaw each session; adjusted the exercises, weight compensation, and maximal active workspace according to each subject's progress; and performed setup and maintenance on the Armeo system. As patients improved, physical therapists would increase the difficulty level and number of repetitions of the games, as well as introduce more difficult games into the training system.

### 2.3. Evaluation

Experimental subjects participated in two types of therapy and were evaluated with functional scales and with upper limb kinematic analysis at pretreatment (T0), post-first treatment (T1), and post-second treatment (T2).

#### 2.3.1. Functional Scales

Clinical data in terms of age at trauma, etiology, and severity were collected at T0 while the Quality of Upper Extremities Skills Test (QUEST), the Melbourne Assessment of Unilateral Upper Limb Function, and the Gross Motor Function Measure (GMFM) were assessed in the clinical examination at T0, T1, and T2.

The Quality of Upper Extremities Skills Test (QUEST) is an internationally validated scale designed to measure treatment outcome in children with upper extremity movement disorders. It explores four domains: dissociated movement, grasp, weight bearing, and protective extension. The dissociated movement domain includes items that counter typical patterns of spastic synergy, representing each joint of the upper limb. Grasp items are based on normal hand grasps as described in developmental literature, arranged in a hierarchical and developmental framework. Weight bearing and protective extension are based on normal developmental sequence and are scored hierarchically based on the degree of abnormality as represented by joint positions. The domain score is a summed-item score converted into a standardized percentage, and the total score is the average of domain scores, with higher scores representing a better quality of movement [22].

The Melbourne Assessment of Unilateral Upper Limb Function, abbreviated to the Melbourne Assessment [23], is a criterion-referenced test developed for use with patients with neurological impairment. The Melbourne Assessment scores the quality of unilateral upper-limb motor function based on items involving reach, grasp, release, and manipulation. In comparison to the Melbourne Assessment, items on the QUEST have been designed also to provide information about postural responses [23, 24].

The GMFM measures the child's overall functional abilities and consists of 88 items, divided into the following sections: (1) lying and rolling, (2) sitting, (3) crawling and kneeling, (4) standing, and (5) walking, running, and jumping. Each section contributes to the total GMFM score [25].

#### 2.3.2. Kinematics

The evaluation consisted of two tasks completed by the subjects, namely horizontal-reaching movements and vertical-reaching movements, while an optoelectronic system for motion capture recorded the kinematics of the participants.

**Figure 2:**
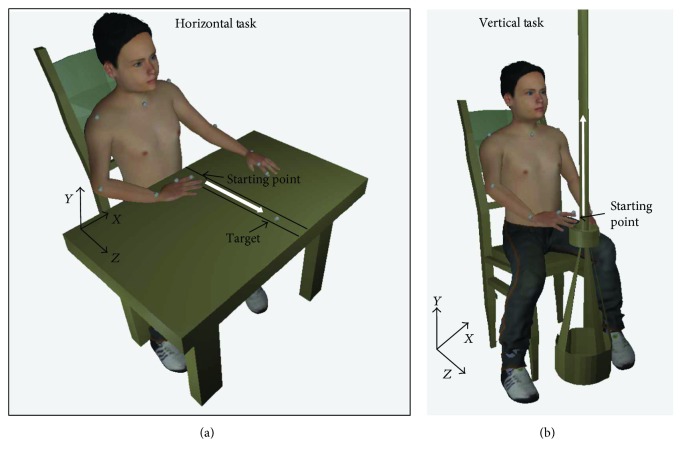
3D sketch of the testing setup, for the horizontal task (left panel) and the vertical task (right panel). Gray dots are the *retroreflective* markers.

*(1) Horizontal Reaching*. During this task, the subject was seated at a table adjusted to a level to support the subject's arms. A stationary marker target was placed on the table along the subject's midline, at a distance equal to 80% of the subject's arm length away from the body. Both hands were to rest on the table, with the hand performing the task to trace the midline path from the body to the marker target and back and the other hand stationary for support (Figure 2(a)). Neither the trunk nor the head were constrained, but the subject was asked to complete the movements as precisely and concisely as possible. The horizontal task was completed three times with each arm by each subject.

*(2) Vertical Reaching*. During the vertical-reaching task (Figure 2(b)), the subject seated comfortably on a chair with a pole with an adjustable support in front of him/her. The pole was adjusted in order to have the edge of the support aligned with the subject's knees. In the starting position, the participant had the tested arm pronated with the finger leaning on the edge of the pole's support. The upper arm was in a neutral adducted position with approximately 90° flexion at the elbow. The participant's other hand was resting on the knee. The subject was asked to move the index finger upward along the pole, following a thin adhesive stripe, as fast as possible but with a maximum precision, reaching the maximum height allowed (but remaining in the seated position) and then returning to the initial position. Neither the trunk nor the head were constrained. The vertical task was completed three times with each arm by each subject.

*(3) Equipment and Kinematic Variables*. The task was recorded using the BTS OEP System (BTS Bioengineering), with 8 cameras with semi-infrared rays that acquire at a frequency of 60 Hz and submultiples. 12 semispherical, retroreflective markers were placed on specific body landmarks: two markers were placed on the trunk—one above vertebrae C7 and the other on the upper part of sternum. Ten markers were placed bilaterally on the acromion, elbow, wrist (ulnar styloid processes), second metacarpal head, and fingernail of the index (Figure 2). The system was able to extrapolate the 3D coordinates of each marker in space and reconstruct the trajectory of each for the whole duration of the movement. Data were analyzed using MATLAB: at first, data were filtered by means of a low-pass filter with a cutoff frequency of 10 Hz. Then, for each movement, three phases were identified, as described in [12]: going phase (Fgo), representing the movement towards the target marker starting from the rest position; adjusting phase (Fadj) that is dedicated to precisely locating the target; and returning phase (Fret) representing the movement towards the starting point. For each phase, many parameters were calculated, both for the horizontal- and the vertical-reaching tasks. In particular, kinematic parameters were divided into three categories and are reported in Table 1:

- *End-point (finger) metrics*: parameters belonging to this category were computed using end-point (finger) kinematic data and provide information about speed of execution, accuracy, efficiency, and smoothness of the movement.
- *Joint kinematics*: angles at the elbow and shoulder were computed as described in previous studies [5, 26]; then, ranges of motion (ROMs) and angular velocity were computed for elbow and shoulder.
- *Trunk compensation*: information derived mainly from the marker placed on the sternum, describing the compensatory movements of the trunk during the reaching movements.

Data were gathered from patients before and after each of the two sets of treatment. Data were divided into groups based on the treatment (namely PT, CIMT, and Armeo) and analyzed comparing treatment type, independently from the order of occurrence of treatments.

### 2.4. Statistical Analysis

Both clinical scale results and the data extracted from the kinematic trials were analyzed in MATLAB using nonparametric statistics, since the Shapiro–Wilk test highlighted a number of data parameters not normally distributed.

To evaluate differences between groups at pretreatment, the Kruskal–Wallis test followed by the Mann–Whitney test with the Bonferroni correction as post hoc analysis were used. Further, the chi-square test was used to check the uniformity of the samples at the beginning of treatment, with regard to sex, etiology, age, distance from event, GCS, and QI. For each treatment, the Wilcoxon test for paired samples was used to compare pre- and posttreatment results, both for kinematic variables and functional scales. To compare treatments, for each variable, the difference (Δ) between post- and pretreatment values was computed, and the Kruskal–Wallis test was used to compare the three treatments, followed by the Mann–Whitney test with the Bonferroni correction as post hoc. For all statistical comparisons, it has defined a maximum value of accepted possible error equal to 5% (*p* = 0.05).

## 3. Results

All children performed the horizontal task and were evaluated with kinematic analysis, while 2 children did not perform the vertical task. 9 subjects (*N* = 9 for horizontal task, *N* = 9 for vertical task) underwent the CIMT rehabilitative treatment, and 9 subjects (*N* = 9 for horizontal task, *N* = 8 for vertical task) completed successfully the Armeo protocol; as 6 patients left the study before its end, only 12 (*N* = 12 for horizontal task, *N* = 11 for the vertical task) subjects were included into the PT group. Specifically, 6 participants out of 9 underwent CIMT as first treatment, 8 patients out of 9 underwent Armeo as first treatment, and 4 patients out of 12 underwent PT as first treatment.

### 3.1. Differences among Groups at Pretreatment

First, a comparison among groups in terms of group features (sex, etiology, age, distance from event, GCS, and QI) and functional evaluations was performed at the beginning of each protocol. No differences were found among groups in terms of group features except for a significant difference (Kruskal–Wallis *p* value = 0.02) in etiology, specifically between Armeo (etiological prevalence of “traumatic brain injury”) and CIMT (etiological prevalence of “hemorrhagic stroke”; Mann–Whitney with Bonferroni correction *p* = 0.02). Furthermore, many significant differences (Kruskal–Wallis *p* value < 0.05) were found in pretreatment functional evaluations among different treatments, mainly in the group that performed Armeo. CIMT and Armeo groups differed in terms of the QUEST-A (80.47 (24.61), 54.69 (12.50), *p* < 0.01), QUEST-C (100 (10), 76 (16), *p* = 0.01), QUEST-tot (70.88 (21.54), 56.63 (7.03), *p* = 0.02), Melbourne Assessment (81.00(13.50), 40.00 (37.00), *p* = 0.02), and GMFM-C (98.00 (2.75), 73.00 (27.75), *p* = 0.03). Moreover, PT and Armeo differed in terms of the QUEST-C (98.00 (12.00), 76.00 (16.00), *p* = 0.03) and Melbourne Assessment (72.00 (17.53), 40.00 (37.00), *p* = 0.03). In contrast, CIMT and PT groups were comparable at the initial evaluation.

**Figure 3:**
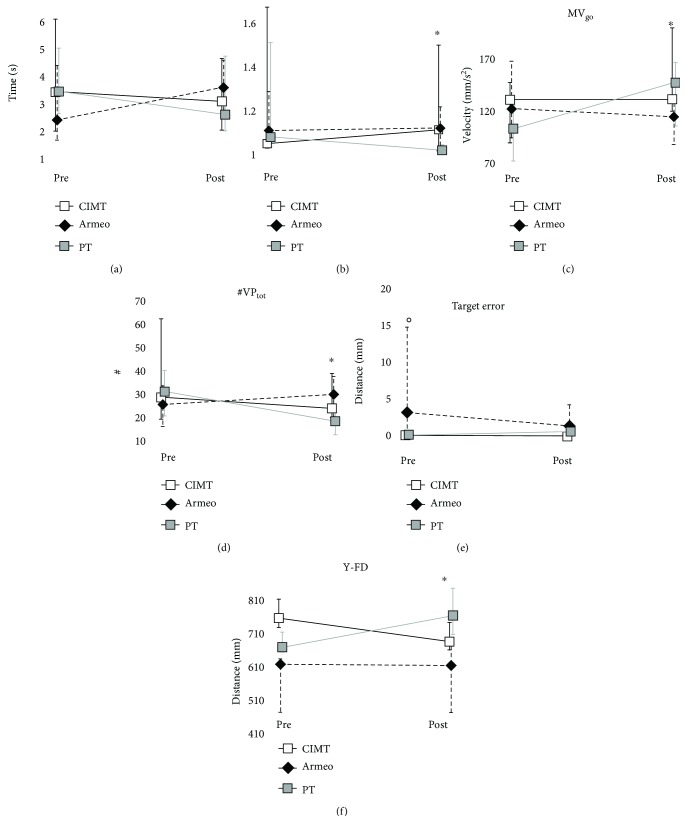
Effect of treatments on end-point metrics. For the horizontal task, pre- and posttreatment values of (a) movement time, (b) hand path ratio during the going phase, (c) mean velocity of the finger during the going phase, (d) total number of velocity peaks, and (e) target error are reported for CIMT (black line), Armeo (black dashed line), and PT (grey line). For the vertical task, pre- and posttreatment values of displacement of (f) the finger along *y*-axis are reported for CIMT (black line), Armeo (black dashed line) and PT (grey line). Data are reported as median (IQR). ^∗^*p* < 0.05 Wilcoxon test, before PT versus after PT; °*p* < 0.05 Mann–Whitney post hoc test at pretreatment, Armeo versus CIMT, Armeo versus PT.

The kinematic data between each group before treatment were also analyzed. For the horizontal task, it was found there was a significant difference in the target error (Figure 3(e)) (Kruskal–Wallis *p* value < 0.01), with Mann–Whitney highlighting lower target error before CIMT (*p* value < 0.01) and PT (*p* value < 0.05) than Armeo.

With regard to the vertical task, a difference in the finger displacement along the *y*-axis (Y-FD) emerged (Kruskal–Wallis *p* value < 0.01), highlighting higher displacement before CIMT than Armeo (Mann–Whitney *p* value < 0.01). In addition, many differences in terms of range of motion emerged, describing a condition characterized by higher functional ranges of motion before CIMT than Armeo, in particular for elbow flex-extension (Kruskal–Wallis *p* value = 0.02, Mann–Whitney *p* value < 0.01), shoulder abduction-adduction (Kruskal–Wallis *p* value < 0.01, Mann–Whitney *p* value < 0.01), and shoulder flex-extension (Kruskal–Wallis *p* value < 0.01, Mann–Whitney *p* value < 0.01).

### 3.2. Changes in Functional Scales Dependent on Treatment

Table 2 shows values of functional scales before and after each treatment. *p* values are the results of the Wilcoxon test for paired data. The table highlights several changes that were conveyed by the CIMT treatment in the QUEST scale (medium effect size, *d* = 0.6) as well as in the Melbourne Assessment (large effect size, *d* = 1.1). Armeo treatment caused improvements in the Melbourne Assessment (large effect size, *d* = 0.9) while the PT protocol in the QUEST scale (large effect size, *d* = 0.9) [27]. No changes in GMFM were detected.

The comparison among the three treatments, considering the variations between post- and pretreatments did not show any significant difference (Kruskal–Wallis test *p* > 0.05).

### 3.3. Changes in Kinematics Dependent on Treatment

For the horizontal task, the improvement of mean angular velocity during shoulder flex-extension was found for CIMT. Several improvements were also found after PT treatment concerning end-point metrics (Figure 3): the significant reductions of HPR_go_ (Figure 3(b)) and #VP_tot_ (Figure 3(d)) as well as an increase of MV_go_ (Figure 3(c)) suggest enhanced efficiency and smoothness of distal movement. Moreover, a trend of improvement was observed with regard to the MT_go_ which decreased after PT (*p* = 0.08, decreases in 8 out of 12 children). With regard to Armeo, no significant differences were found between pre- and posttreatments with regard to end-point metrics; in contrast, a significant but small (i.e., 0.52 degrees) reduction of ROM of shoulder flex-extension emerged (reduces in 8 out of 9 children). No significant differences emerged with regard to the compensatory movement of the trunk after CIMT, Armeo, and PT. Pre-/post values of kinematic parameters extracted for the horizontal task for each treatment are shown in Table 3.

With regard to the vertical task, no significant differences emerged for CIMT and Armeo treatments between pre- and postevaluations; on the contrary, some improvements emerged after PT, in particular an increase of Y-FD (Wilcoxon *p* value = 0.02) (Figure 3(f)) and of the mean angular velocity of the elbow flex-extension (Wilcoxon *p* value = 0.03). Trends of improvement, even if not significant, were observed: with regard to compensatory movements of the trunk, a reduction of trunk lateral bending emerged after CIMT (X-TD_go_: *p* = 0.05); also, a trend of increase of movement efficiency was observed after Armeo (HPR_go_: *p* = 0.08, HPR_go_ increases in 6 children out of 8), and after PT, increase of shoulder abduction-adduction ROM (*p* = 0.05, increase in 9 out of 11 children) was observed. Pre-/post values of kinematic parameters extracted for the vertical task for each treatment are shown in Table 4.

The comparison of variations (post-pre) among the three treatments highlighted a significant difference in ROM of the shoulder during flex-extension (Kruskal–Wallis *p* = 0.02) that decreased after Armeo while it increased after CIMT (Mann–Whitney *p* = 0.03) and a significant difference in the mean angular velocity of the shoulder in flex-extension during the going phase (Kruskal–Wallis *p* = 0.01) that increased after CIMT while it decreased after PT (Mann–Whitney *p* = 0.01).

For the vertical task, a difference between groups emerged with regard to the modification of the ROM of shoulder abduction-adduction during the going phase (Kruskal–Wallis *p* = 0.03) that increased after PT while it was reduced after Armeo (Mann–Whitney *p* = 0.05). Also, compensatory movements of the trunk, namely X-TD_go_ (Kruskal–Wallis *p* = 0.03), were reduced after CIMT while they increased after PT (Mann–Whitney *p* = 0.04).

## 4. Discussion

In children affected by acquired hemiplegia, several treatments were developed to improve functional recovery of the upper limbs.

In hemiplegic patients, the rehabilitation of the upper limb skills is crucial, because the recovery has an immediate impact on patient quality of life. Recent literature documents demonstrate the great effort made by rehabilitation facilities to enhance the recovery of motor function [28]. Two of the most widely used rehabilitation methods used for this aim are CIMT (Constraint-Induced Movement Therapy) and robot-aided therapy. The plurality of treatments necessitates a comparison of the effectiveness of each; in addition, the current lack of availability of quantitative methods makes the use of objective measures of functional recovery essential. Next to the functional scales, the clinic has recently introduced methods of kinematic analysis of the upper limb movement to get more objective and quantifiable measures.

In this study, we evaluated a group of children and adolescents with upper limb movement impairment after ABI that underwent intensive rehabilitation treatment. The rehabilitation program included two consecutive four-week periods of treatment, attended in a random order: one period of physical therapy (PT) and one period of a rehabilitative treatment randomly chosen between Constraint-Induced Movement Therapy (CIMT) and training with the exoskeleton Armeo®Spring. The aim of this study was to compare the effect of different types of treatments on functional abilities in a group of children and adolescents suffering from ABI. Clinical functional scales and upper limb kinematics data were used as assessment tools.

Only a very low number of studies have previously quantified recovery of upper limbs in children after ABI; our study investigated a very wide perspective in terms of movement (kinematic data), gross motor performance, and hand function. Also, attention was focused on both clinical-functional scales and kinematic data during different types of treatment.

In terms of functional scale, the most significant improvements were obtained after CIMT. In fact, this treatment demonstrated progress in both the QUEST and Melbourne Assessment. The other treatments produced significant changes in a single assessment scale: Armeo modified the Melbourne Assessment score and thus increased the quality of the upper limb movement, while PT produced a significant improvement in the QUEST scale, in particular in postural responses. All these improvements had a large effect size, except for the QUEST scale in the CIMT group.

Therefore, CIMT seems to still be the most effective treatment as evidenced by the literature of the past 20 years [29], significantly improving both the quality of motor limb function (analyzed by the Melbourne Assessment) and postural responses and selective motility (evaluated by the QUEST) [30].

The kinematic analysis of movements during horizontal as well as vertical tasks showed several improvements in terms of efficiency and smoothness of end-effector movements and elbow angular velocity after PT, suggesting its positive influence on distal movements. In contrast, CIMT conveyed more changes at the shoulder and trunk districts that means improvements of proximal kinematics and reduction of compensatory movements. An improvement of the movement efficiency was observed after Armeo treatment: specifically, not only a trend of increase of the hand-path ratio during the vertical reaching but also a significant reduction of the shoulder flex-extension in the horizontal task was observed. Compared with other treatments, Armeo showed an improvement of shoulder abduction-adduction during the vertical task (i.e., a reduction of compensatory movements of the shoulder). These opposing results may be due to the mechanical constraints that the exoskeleton gives during therapy.

More generally, PT seems to be more effective in terms of kinematic variations than CIMT and Armeo: a possible hypothesis is that, since PT was often provided as a second treatment, this depends on a sort of summation effects of the two treatments. Moreover, it has to be noted that the PT sample size is bigger than the other treatments. Compared to a previous work [12], our data after CIMT showed smaller improvements; this may depend on the duration of the treatment, that is, 4 weeks in the current manuscript versus 10 weeks in the work by Cimolin and coworkers. The results of the present study are in line with those by Cope and collaborators [31] where there were improvements of the functional scales but just few trends of improvement with regard to the kinematics after a 2-week rehabilitation with CIMT in children with hemiplegia.

With respect to the treatment with Armeo, the small number of significant improvements can be attributed to the fact that patients who were assigned to this type of treatment were on average more functionally compromised at the beginning of their rehabilitative path, with an important limitation of the upper limb distal level. Specifically, they had worse functional abilities at the hand level and lower functional ranges of motion at joint levels. It is already known, indeed, that patients with a moderate degree of impairment seem to benefit the most [31].

None of the three treatments changed the gross motor skills (no significant changes in GMFM) because the patients recruited for the upper limb treatment, generally, had a framework of global consolidated skills and the rehabilitation was more concerned with the functionality of the upper limb and not with gross motor abilities.

The kinematic data allow us to make objective measurements of the movement characteristics of patients after ABI. Kinematic analysis, known in the literature especially for gait analysis, proves to be able to describe very well the upper limb movements and is a valuable aid in discriminating with greater precision the modifications that each treatment cause.

This study has some limitations. The small number of participants resulted in limited strength with regard to the statistical findings. A larger sample could provide the opportunity to make a deeper investigation of the differences between the treatments. In addition, with a larger group of children, it would be possible to investigate whether the improvements are greater in patients who start the treatment closer to the time of their injury, by comparing the results of the program between children with shorter and longer postinjury times. A bigger sample would also allow to evaluate the effects on functional abilities of different matching of treatments.

Another critical point concerns the group of patients who received robotic treatment with Armeo. They had overall worse limb function when beginning treatment than the other patients and even than other groups performing this treatment [10, 11], and this could have determined the lack of improvement in kinematic data.

Despite these limitations, the present study has interesting clinical implications: from the rehabilitation point of view, this study allowed the development of assessment and treatment protocols that can be used for all patients with ABI that undergo rehabilitation treatments aimed at improving the use of the upper limbs. Further, the results of this study also allow us to give more precise information about the type of treatment to be offered to children suffering from hemiplegia from ABI. In fact, we can choose the treatments after identifying the target that one wants to reach in the single patient, for example, improve the quality of unilateral upper-limb motor function or increase postural responses. Moreover, in some patients, the integration of multiple treatments will be indicated because they are complementary and not only differently effective.

In conclusion, this study investigated the effects of different types of upper-limb rehabilitative treatments on the functional improvement of children and adolescents with ABIs. It was found that CIMT treatment is overall the most effective in terms of quality in motor limb function and postural responses as evidenced by functional scales, while physical therapy and robot exoskeleton-aided therapy convey improvements only in the QUEST and Melbourne Assessment, respectively. Kinematic analysis results suggest that CIMT is able to foster proximal movement improvements, in particular at the shoulder joint. In the contrary, PT showed good results in terms of distal movements, including increase of finger speed and fluidity. Finally, Armeo treatment conveyed improvements in the shoulder performing the vertical but also a reduction of its functionality in the horizontal one. These data suggest that these treatments are complementary and that it would be important to offer to hemiplegic children a combination of these protocols depending on the main rehabilitative goal. Future works will investigate the ability to prescribe specific treatments in order to maximize patient improvements.

## Figures and Tables

**Table 1:** Kinematic variables computed for the horizontal and vertical task.

		Horizontal task	Vertical task
*End-point (finger) metrics*			
Hand path ratio (P_go_) HPR_go_	A measure of how directly the hand moves toward the target, computed as the ratio between the length of the real subject's hand (finger) path and the length of the theoretical or desired trajectory. This metric quantifies the movement efficiency.	✓	✓
Displacement along *y*-axis, Y-FD	The difference between the maximum and the minimum *y* coordinate during the whole movement, representing the vertical displacement of the finger.		✓
Movement time (P_go_) MT_go_	Time from the onset to the offset of the going phase quantifies the movement speed and upper extremity function within the given task.	✓	
Target error	It is a measure of the movement quality in terms of accuracy, computed as the maximum distance from the index finger to the target during the adjusting phase.	✓	
Mean velocity (P_go_) MV_go_	Mean arm velocity attained during the going phase computed from the speed profile of the finger.	✓	✓
Number of velocity peaks (Tot), #VP_tot_	It is a quality measure of the movement smoothness computed from the speed profile of the finger during the whole movement.	✓	✓
*Joint kinematics*			
Joint ROMs	Range of motion for the elbow (flex-extension) and the shoulder (abduction-adduction and flex-extension) computed as the difference between the maximum and the minimum angle, considering the whole movement.	✓	✓
Mean angular velocity (MAV)	Mean angular velocity during elbow flex-extension and shoulder abduction-adduction or flex-extension, during the going phase.	✓	✓
*Trunk compensation*			
Trunk 3D path	3D path length of the marker placed on the sternum	✓	✓
Displacement along *z*-axis (P_go_), Z-TD_go_	Displacement of the marker placed on the sternum along the *z*-axis (towards the target) during the going phase. It quantifies trunk flexion.	✓	
Displacement along *x*-axis (P_go_), X-TD_go_	Displacement of the marker placed on the sternum along the *x*-axis during the going phase. It quantifies trunk lateral bending.		✓

**Table 2:** Median (IQR) of functional scales before (pre) and after (post) each treatment. The sample size is *n* = 8 for the CIMT, *n* = 9 for the Armeo, and *n* = 11 for the PT groups. *P* values refer to Wilcoxon test. Bold values: *p* < 0.05.

	Pretreatment	Posttreatment	*p* value
CIMT (*n* = 8)			
QUEST-tot	70.88 (21.54)	82.02 (17.10)	**0.03**
QUEST-A	80.47 (24.61)	86.72 (21.48)	**0.04**
QUEST-B	77.78 (15.74)	77.78 (17.59)	0.11
QUEST-C	100.00 (10.00)	100.00 (3.00)	0.18
QUEST-D	59.73 (30.56)	59.73 (25.00)	0.18
Melbourne Assessment %	81.00 (13.50)	82.50 (7.25)	**0.02**
GMFM total	214.00 (20.50)	249.00 (18.00)	0.07
Armeo (*n* = 9)			
QUEST-tot	56.63 (7.03)	56.63 (9.43)	0.07
QUEST-A	54.69 (12.50)	54.69 (15.63)	0.11
QUEST-B	55.56 (14.81)	55.56 (14.81)	0.32
QUEST-C	76.00 (16.00)	80.00 (16.00)	0.11
QUEST-D	41.67 (8.33)	41.67 (8.33)	0.08
Melbourne Assessment %	40.00 (37.00)	43.00 (26.00)	**0.03**
GMFM total	204.50 (28.25)	214.00 (21.75)	0.07
PT (*n* = 11)			
QUEST-tot	70.49 (14.81)	73.36 (18.95)	**0.01**
QUEST-A	70.31 (17.97)	76.56 (14.85)	**0.02**
QUEST-B	70.37 (16.67)	77.78 (20.37)	0.89
QUEST-C	98.00 (12.00)	100.00 (12.00)	0.10
QUEST-D	44.40 (13.89)	52.78 (19.45)	**0.04**
Melbourne Assessment %	72.00 (17.53)	77.00 (18.50)	0.14
GMFM total	240.00 (28.50)	240.50 (26.25)	0.07

**Table 3:** Kinematic variables before (pre) and after (post) each treatment for the horizontal task. Data are presented as median (IQR). The sample size is *n* = 9 for the CIMT, *n* = 9 for the Armeo, and *n* = 12 for the PT groups. *p* values refer to the Wilcoxon test. Bold values: *p* < 0.05.

	Horizontal task parameters	Pretreatment	Posttreatment	*p* value
CIMT (*n* = 9)				
End-point metrics	MT_go_ [s]	3.42 (4.08)	3.08 (2.62)	0.25
	HPR_go_	1.05 (0.64)	1.11 (0.48)	0.73
	MV_go_ [mm/s]	95.74 (60.63)	111.72 (49.20)	0.50
	#VP_tot_	28.67 (43.00)	24.00 (19.00)	0.16
	Target error	0 (0)	0 (0)	0.88
Joint kinematics	ROM shoulder flex-ext [°]	45.82 (5.35)	47.57 (12.46)	0.25
	ROM shoulder abd-add [°]	13.54 (2.49)	19.32 (19.32)	0.10
	ROM elbow flex-ext [°]	47.55 (21.26)	44.37 (14.17)	0.91
	MAV shoulder flex-ext [°/s]	3.92 (4.52)	7.16 (5.95)	**<0.01**
	MAV shoulder abd-add [°/s]	12.70 (7.95)	14.19 (7.31)	0.65
	MAV elbow flex-ext [°/s]	13.49 (6.21)	18.12 (9.45)	0.16
Trunk	Trunk 3D path	39.12 (21.06)	34.29 (16.60)	0.91
	Z-TD_go_	90.11 (78.62)	74.94 (42.20)	0.65
Armeo (*n* = 9)				
End-point metrics	MT_go_ [s]	2.41 (2.73)	3.59 (2.10)	0.50
	HPR_go_	1.11 (0.26)	1.12 (0.18)	0.73
	MV_go_ [mm/s]	122.18 (73.32)	114.65 (39.97)	0.43
	#VP_tot_	25.67 (17.33)	30.00 (20.72)	0.91
	Target error	3.14 (14.44)	1.30 (3.91)	0.36
Joint kinematics	ROM shoulder flex-ext [°]	45.52 (9.59)	45.00 (15.86)	**0.01**
	ROM shoulder abd-add [°]	13.61 (7.40)	16.13 (4.72)	0.20
	ROM elbow flex-ext [°]	31.52 (14.30)	27.27 (16.73)	0.65
	MAV shoulder flex-ext [°/s]	6.68 (6.59)	6.03 (8.17)	0.72
	MAV shoulder abd-add [°/s]	15.42 (9.90)	13.37 (4.77)	0.16
	MAV elbow flex-ext [°/s]	14.44 (9.91)	9.63 (9.44)	0.73
Trunk	Trunk 3D path	352.52 (195.01)	443.60 (189.82)	0.73
	Z-TD_go_	136.16 (44.51)	158.63 (54.40)	1.00
PT (*n* = 12)				
End-point metrics	MT_go_ [s]	3.44 (2.38)	2.61 (2.73)	0.08
	HPR_go_	1.08 (0.47)	1.02 (0.03)	**0.03**
	MV_go_ [mm/s]	103.36 (53.40)	147.31 (61.05)	**0.04**
	#VP_tot_	31.17 (19.50)	18.50 (9.25)	**0.02**
	Target error	0.10 (0.26)	0.67 (0.53)	0.31
Joint kinematics	ROM shoulder flex-ext [°]	44.95 (17.62)	43.23 (8.31)	0.97
	ROM shoulder abd-add [°]	17.73 (8.68)	12.82 (14.56)	0.42
	ROM elbow flex-ext [°]	41.45 (19.07)	39.57 (28.90)	0.57
	MAV shoulder flex-ext [°/s]	5.88 (8.31)	5.42 (3.57)	0.52
	MAV shoulder abd-add [°/s]	11.68 (3.76)	16.72 (14.28)	0.11
	MAV elbow flex-ext [°/s]	12.82 (8.81)	13.85 (7.39)	0.42
Trunk	Trunk 3D path	313.42 (186.79)	300.32 (182.32)	0.18
	Z-TD_go_	90.01 (102.48)	108.07 (91.19)	0.85

**Table 4:** Kinematics variables before (pre) and after (post) each treatment for the vertical task. Data are presented as median (IQR). The sample size is *n* = 9 for the CIMT, *n* = 8 for the Armeo, and *n* = 11 for the PT groups. *p* values refer to Wilcoxon test. Bold values: *p* < 0.05.

	Vertical task parameters	Pretreatment	Posttreatment	*p* value
CIMT (*n* = 9)				
End-point metrics	HPR_go_	1.10 (0.89)	1.09 (0.61)	0.09
	Y-FD [mm]	757.14 (84.78)	686.79 (81.75)	0.30
	MV_go_ [mm/s]	150.57 (47.00)	209.59 (76.55)	0.65
	#VP_tot_	48.00 (18.67)	42.67 (39.00)	0.34
Joint kinematics	ROM shoulder flex-ext [°]	116.56 (22.72)	106.45 (38.87)	0.15
	ROM shoulder abd-add [°]	126.69 (15.41)	126.20 (35.95)	1.00
	ROM elbow flex-ext [°]	56.61 (11.46)	62.26 (18.99)	0.31
	MAV shoulder flex-ext [°/s]	22.43 (16.58)	25.06 (26.00)	0.84
	MAV shoulder abd-add [°/s]	18.74 (13.24)	19.66 (8.48)	0.46
	MAV elbow flex-ext [°/s]	15.59 (8.19)	18.50 (6.62)	0.74
Trunk	Trunk 3D path	477.38 (103.88)	386.72 (157.66)	0.20
	X-TD_go_	58.35 (25.96)	49.77 (18.30)	0.05
Armeo (*n* = 8)				
End-point metrics	HPR_go_	1.43 (0.39)	1.26 (0.14)	0.08
	Y-FD [mm]	618.80 (160.00)	615.27 (217.03)	0.84
	MV_go_ [mm/s]	125.76 (107.67)	152.51 (66.25)	1.00
	#VP_tot_	46.46 (26.83)	35.67 (32.83)	0.31
Joint kinematics	ROM shoulder flex-ext [°]	66.93 (34.99)	69.95 (41.34)	0.55
	ROM shoulder abd-add [°]	54.39 (48.41)	56.59 (24.32)	0.31
	ROM elbow flex-ext [°]	38.32 (11.65)	35.09 (10.73)	0.64
	MAV shoulder flex-ext [°/s]	13.96 (16.62)	9.56 (9.38)	0.15
	MAV shoulder abd-add [°/s]	13.85 (10.24)	14.05 (8.53)	0.46
	MAV elbow flex-ext [°/s]	8.78 (6.28)	10.49 (5.33)	0.74
Trunk	Trunk 3D path	351.34 (58.49)	303.72 (130.18)	0.46
	X-TD_go_	46.51 (25.83)	52.57 (11.13)	0.55
			
PT (*n* = 11)				
End-point metrics	HPR_go_	1.15 (0.49)	1.12 (0.48)	0.20
	Y-FD [mm]	669.71 (58.56)	764.82 (138.49)	**0.02**
	MV_go_ [mm/s]	185.59 (106.09)	194.79 (41.96)	0.52
	#VP_tot_	56.00 (42.33)	36.00 (32.42)	0.97
Joint kinematics	ROM shoulder flex-ext [°]	95.13 (36.11)	99.75 (16.26)	0.32
	ROM shoulder abd-add [°]	103.43 (67.88)	121.15 (18.94)	0.05
	ROM elbow flex-ext [°]	57.71 (23.32)	62.54 (15.12)	0.43
	MAV shoulder flex-ext [°/s]	26.50 (20.66)	22.46 (13.69)	0.40
	MAV shoulder abd-add [°/s]	18.99 (14.07)	18.21 (10.20)	0.58
	MAV elbow flex-ext [°/s]	16.36 (4.14)	18.98 (5.86)	**0.03**
Trunk	Trunk 3D path	347.89 (155.03)	348.67 (180.99)	0.17
	X-TD_go_	49.77 (8.77)	66.22 (24.72)	0.10
